# Spectrum and carrier frequency of DMD in Yueyang, China: a population-based analysis using NGS and MLPA

**DOI:** 10.3389/fgene.2026.1889156

**Published:** 2026-09-03

**Authors:** Qun Zhu, Ang Sun, Yuyao Zu, Yan Zeng, Liya Chen, Yuan Yang, Zhen Liu, Yuting Sun, Xuzhen Huang, Xiaobu Shen

**Affiliations:** 1 Key Laboratory of Birth Defects Prevention and Control of Yueyang City, Yueyang, Hunan, China; 2 Department of Medical Genetics, Yueyang Maternal and Child Health Hospital, Yueyang, Hunan, China; 3 Technical Enablement Center, Zhejiang Biosan Bioscience Co., Ltd., Hangzhou, Zhejiang, China

**Keywords:** carrier frequency, copy number variants, duchenne muscular dystrophy, mutation spectrum, prenatal carrier screening, variants of uncertain significance

## Abstract

**Background:**

Duchenne muscular dystrophy (DMD) is an X-linked recessive disorder caused by mutations in the *DMD* gene. Understanding the carrier frequency and mutation spectrum in specific populations is critical for genetic counseling and early intervention. However, data on DMD carrier frequency among women of childbearing age and early pregnancy in Yueyang City, China, remain limited. This study aimed to characterize the carrier rate and mutation profile to support preventive strategies and reduce disease incidence.

**Methods:**

A total of 25,611 women of childbearing age or early pregnancy from Yueyang City were enrolled. Combined next-generation sequencing and multiplex ligation-dependent probe amplification were used to detect pathogenic/likely pathogenic (P/LP) variants, copy number variants (CNVs), and small indels. Variants were classified per established guidelines. Carrier rates and geographical distribution were analyzed. Prenatal diagnosis was offered to identified carriers with follow-up to assess outcomes.

**Results:**

Twenty-eight women were identified as P/LP carriers (0.11%), representing 25 distinct variants. CNVs constituted the majority (71.43%), with exon 45–55 deletions (64.29%) predominating over duplications (7.14%); notably, 13/18 CNVs clustered in this hotspot. SNVs and small indels accounted for the remaining 28.57%. Intra-regional variation was marked, with the highest rate in Yunxi District (0.74%). Additionally, 81 VUSs (51 distinct types) were detected, 66.67% being missense. One male fetus inheriting a maternal VUS developed DMD-like features postpartum. Overall, 12 variants (1 LP, 11 VUSs) were previously unreported, including a nonsense variant c.3502G>T (p.E1168*) classified as LP.

**Conclusion:**

This first population-based study in Yueyang City, China, characterized the DMD carrier frequency (0.11%) and mutation spectrum among women of childbearing age or in early pregnancy. It revealed geographical heterogeneity and a high prevalence of CNVs, especially exon 45–55 deletions. Crucially, it highlights the underappreciated screening value of VUS. We recommend focused attention on VUS, particularly those with Bayesian scores ≥3, in genetic counseling and prenatal diagnosis to improve preventive strategies and reduce DMD incidence.

## 1 Introduction

Duchenne muscular dystrophy (DMD) is an X-linked recessive genetic disorder and the most common form of childhood muscular dystrophy, with an incidence ranging from 1 in 3,600 to 1 in 5,000 (Thapa et al., 2023). Typically, male patients exhibit clinical symptoms between the ages of 3 and 5 years; these symptoms progressively worsen into severe muscle weakness over several years, ultimately leading to loss of ambulation (Gatto et al., 2024). The majority of affected individuals succumb to respiratory failure or cardiomyopathy (Sheikh and Yokota, 2021). Males carrying pathogenic (P) or likely pathogenic (LP) variants in the *DMD* gene may be affected; in contrast, females with such variants are considered carriers (Chen et al., 2021). Female DMD carriers can manifest a range of clinical symptoms. Therefore, it is essential to conduct early screening for those with reproductive needs and to identify infants at risk of symptom development (Laura Carraro Arianna Iosca et al., 2023).

Because the most common pathogenic mutations in DMD are large exon deletions or duplications, Multiplex Ligation-dependent Probe Amplification (MLPA) remains the preferred initial method for detection (Zinina et al., 2022). Although MLPA can also identify small indels (insertions/deletions <20 bp) in some cases, it cannot detect single-nucleotide variants (SNVs) or deep intronic mutations (Guevara-Fujita et al., 2021). While Next-Generation Sequencing (NGS) enables comprehensive screening of all variant types, its accuracy in GC-rich or repetitive genomic regions may require optimization (Zhang et al., 2019). Consequently, for DMD screening, combining initial NGS with subsequent MLPA verification proves more cost-effective than relying solely on NGS.

This retrospective study analyzed NGS and MLPA results from 25,611 subjects at the Prenatal Diagnosis Center of Yueyang Maternal and Child Health Hospital. We aimed to summarize carrier rates and the detection of deletions/duplications, SNVs and indels in the DMD gene, with a specific focus on identifying variants of uncertain significance (VUS). Additionally, phenotypes and prenatal diagnosis outcomes of P/LP variant carriers were followed up to provide comprehensive data for genetic counseling. Furthermore, this research aims to raise female carriers’ health awareness and highlight potential fetal risks.

## 2 Materials and methods

### 2.1 Subjects

**FIGURE 1:**
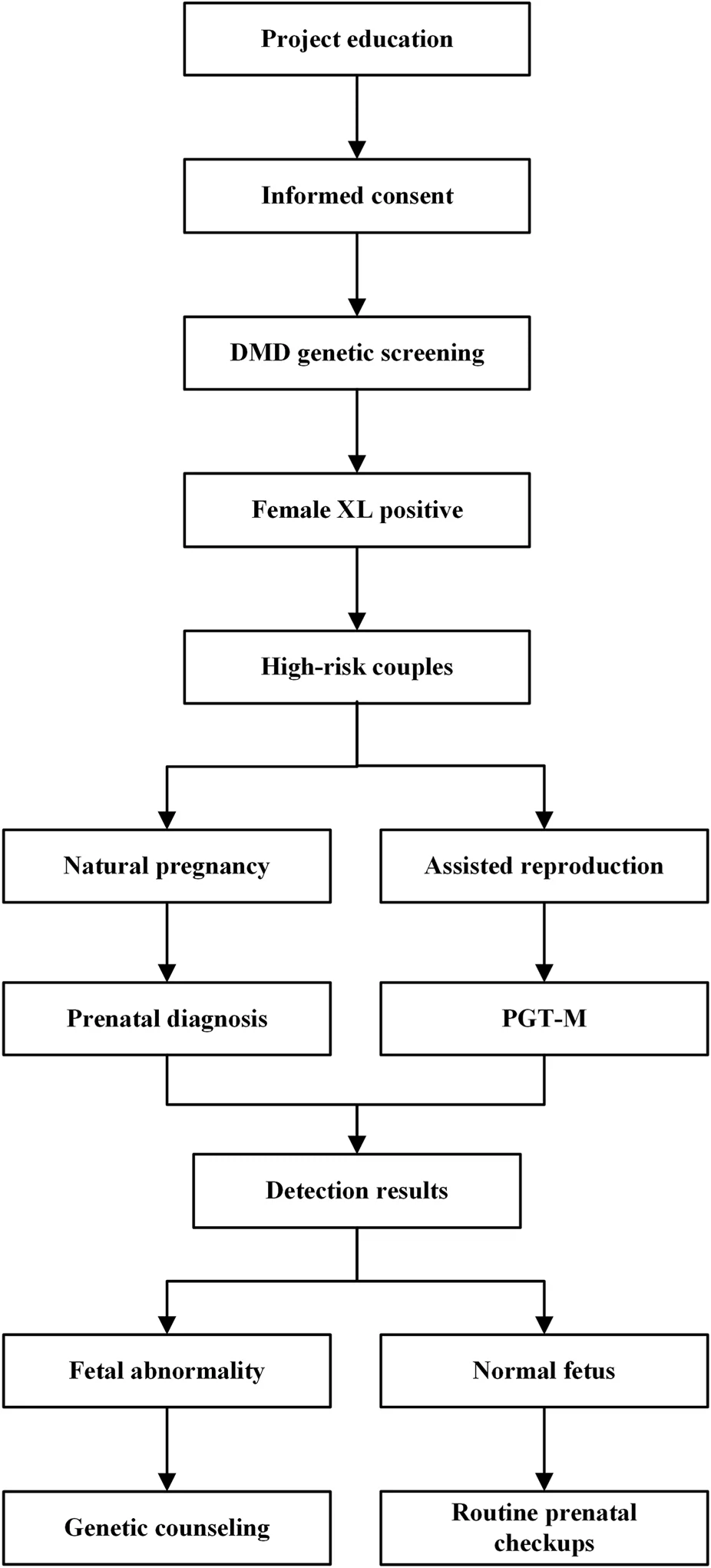
DMD carrier screening flowchart. This flowchart outlines the process for identifying high-risk couples and their reproductive options following DMD carrier screening. Female carriers of a DMD variant (Female XL Positive) and their partners are defined as high-risk couples. Two pathways are then available: natural pregnancy with prenatal diagnosis, or assisted reproduction with preimplantation genetic testing for monogenic disorders (PGT-M). Outcomes lead to genetic counseling for abnormal results or routine prenatal care for a normal fetus. Abbreviations: PGT-M, preimplantation genetic testing for monogenic disorders; XL, X-linked.

From January to December 2024, 25,611 participants underwent DMD gene screening at Yueyang Maternal and Child Health Hospital. Inclusion criteria were: (1) couples planning conception or individuals with singleton pregnancies ≤13^+6^ weeks’ gestation; (2) provision of written informed consent. Exclusion criteria were: (1) individuals undergoing assisted reproductive technology (ART); (2) those diagnosed with monogenic disorders or identified as carriers; (3) recipients of blood transfusions, organ transplants, or immunotherapy within the preceding year. The DMD carrier screening flowchart is shown in Figure 1.

Ethical approval (protocol code 2025015) was obtained from the Ethics Committee of Yueyang Maternal and Child Health Hospital, and all research was performed in accordance with relevant guidelines/regulations. Written informed consent was obtained from all participants before enrollment.

### 2.2 Methods

#### 2.2.1 NGS method

Genomic DNA was extracted from samples using a commercial kit (Bioer, China), followed by purification. Subsequently, DNA fragmentation and end repair were performed with an enzyme cocktail (GeneMeta, China), and adapters were ligated to construct libraries through amplification and size selection. Target regions were enriched via hybrid capture using magnetic beads (iGeneTech, China), completing final library preparation. Libraries were sequenced on the DNBSEQ-T7 platform (MGI, China) with 150-bp paired-end reads.

Key NGS quality metrics-including Q30 score (>75%), average sequencing depth (>200×), and the percentage of target regions covered at ≥20× depth (20×%, > 98%)-were evaluated to ensure the reliability and accuracy of SNV detection. All metrics met predefined empirical thresholds, confirming high-quality data suitable for downstream variant calling.

Raw reads were trimmed and filtered (fastp v1.0.1), aligned to the human reference genome GRCh37 (hg19) using BWA-MEM, and the resulting Binary Alignment Map (BAM) files were sorted and indexed (SAMtools v1.18). Duplicate reads were marked and base quality score recalibration (BQSR) was performed using the Genome Analysis Toolkit (GATK). GATK HaplotypeCaller generated raw Variant Call Format (VCF) files for SNVs/indels. Copy number variants (CNVs) were called using Biosan’s depth-based algorithm: after read-depth normalization, control samples from a fixed reference set were selected, producing raw VCF files. Both VCF sets were filtered by quality metrics and allele frequency to remove false positives. SNV/indel variants were annotated with the Ensembl Variant Effect Predictor (VEP); CNVs with AnnotSV and BEDTools. Variant interpretation was performed following the 2015 guidelines of the American College of Medical Genetics and Genomics (ACMG) and the Association for Molecular Pathology (AMP) (Richards et al., 2015). A Bayesian framework integrating ACMG/AMP criterion weights was used to derive a continuous score (range 0–5); variants with a score ≥3 were designated as DMD-VUS of concern.

#### 2.2.2 MLPA method

Genomic DNA was extracted using a commercial kit (Bioer, China) and purified. Subsequently, the DNA was denatured at 95 °C to generate single strands. Probes for the DMD gene (MRC-Holland, Netherlands) were hybridized to target regions. The dual-channel combinatorial MLPA probe kit, comprising P034 DMD Mix 1 and P035 DMD Mix 2, is specifically designed to detect CNVs-including exon deletions and duplications-in the human DMD gene. The two probe mixes collectively cover all 79 exons of the *DMD* gene: P034 DMD Mix 1 targets exons 1–10, 21–30, 41–50, and 61–70, whereas P035 DMD Mix 2 targets exons 11–20, 31–40, 51–60, and 71–79. This complementary and non-overlapping coverage design minimizes the risk of false-negative results resulting from incomplete exon interrogation. Upon hybridization of adjacent probes to contiguous sequences, ligation was conducted using a thermostable ligase (MRC-Holland). Capillary electrophoresis was performed on an Applied Biosystems 3500xL Dx Genetic Analyzer, and fluorescence signals corresponding to specific amplicons were recorded. Coffalyser.Net software converted raw signal data into electropherograms. Through peak-height normalization against reference samples, we analyzed variations in the test samples.

#### 2.2.3 Follow-up

A follow-up study was initiated for women with confirmed DMD carrier status. The primary objective was to document their subsequent reproductive choices, specifically (1) the uptake of PGT-M to prevent transmission, or (2) the pursuit of prenatal diagnostic procedures after natural conception. (3) In parallel, a secondary objective involved the systematic clinical evaluation of the carriers themselves and their offspring to identify and document any potential DMD-associated phenotypes.

#### 2.2.4 Statistical analysis

All statistical analyses were performed using WPS Office (version 2025; Kingsoft Corporation, Beijing, China). And comparisons for calculating sensitivity and specificity were performed using statistical software SPSS version 19.0 (IBM Corp., Armonk, NY, USA). The percentage for each categorical variable was calculated as the number of observed cases divided by the total number of valid observations, multiplied by 100% (n/N) × 100%.

## 3 Results

### 3.1 Screening results

**FIGURE 2:**
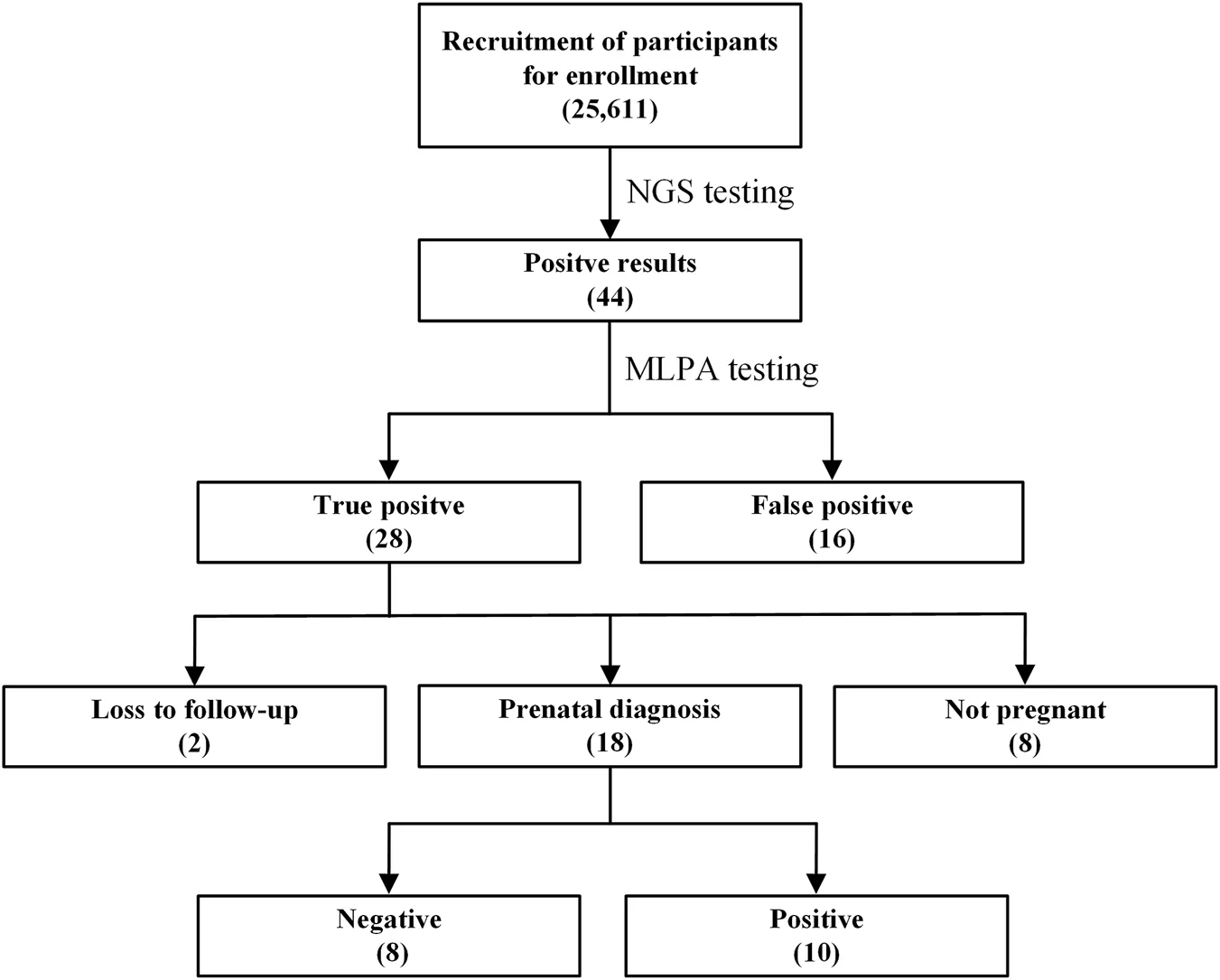
Among 25,611 participants, 44 screened positive by NGS. MLPA validation confirmed 28 true positives. Follow-up of these cases revealed that 18 carriers underwent prenatal diagnosis, which identified 10 affected fetuses.

We conducted DMD carrier screening on 25,611 samples. By NGS testing, 44 positive results were detected; among which 16 were confirmed as false positives after MLPA verification. Among these, 28 female carriers were identified, yielding a positive rate of 0.11%. This finding aligns with the X-linked recessive inheritance pattern of DMD. Detailed genetic screening results are summarized in Table 1 and Figure 2.

**TABLE 1:** DMD carrier-positive samples.

Sample ID	Maternal age (years)	Sample type	Gestational age (weeks)	Variant position	Heterozygosity	ACMG pathogenicity
1	34	PB	0	E45-49dup	het	LP
2	43	PB	13	E68-71del	het	P
3	32	PB	10^+6^	c.3502G>T (p.E1168*)	het	LP
4	22	PB	12^+3^	E3-7del	het	P
5	36	PB	16^+1^	c.1812 + 1G>A	het	LP
6	29	PB	7	c.4300A>T (p.K1434*)	het	LP
7	24	PB	14^+2^	E46-54del	het	LP
8	27	PB	8^+3^	c.10231dup (p.T3411Nfs*22)	het	LP
9	30	PB	8	E56-62del	het	P
10	27	PB	12^+2^	E48-49del	het	P
11	29	PB	10^+3^	E45-55del	het	P
12	26	PB	9^+6^	E46-52del	het	P
13	27	PB	7^+4^	E51-60dup	het	LP
14	36	PB	7^+1^	c.10454del (p.L3485Rfs*11)	het	P
15	20	PB	10^+2^	E48-55del	het	P
16	24	PB	16	E50-59del	het	LP
17	25	PB	12^+4^	E48del	het	P
18	37	PB	0	E48-49del	het	P
19	39	PB	0	E48-53del	het	LP
20	43	PB	0	E51-52del	het	LP
21	30	PB	4^+6^	c.1615C>T (p.R539*)	het	P
22	28	PB	12	E48del	het	P
23	24	PB	10^+5^	E54-62del	het	P
24	25	PB	0	E30-44del	het	LP
25	21	PB	0	c.9100C>T (p.R3034*)	het	P
26	29	PB	0	E45del	het	LP
27	35	PB	14^+2^	E51-52del	het	LP
28	31	PB	12^+3^	c.5287C>T (p.R1763*)	het	P

ACMG, American College of Medical Genetics and Genomics. E, exon. Del, deletion. Dup, duplication. Het, Heterozygote. LP, likely pathogenic. P, pathogenic. PB, peripheral blood.

### 3.2 Distribution of positive DMD cases by region

**FIGURE 3:**
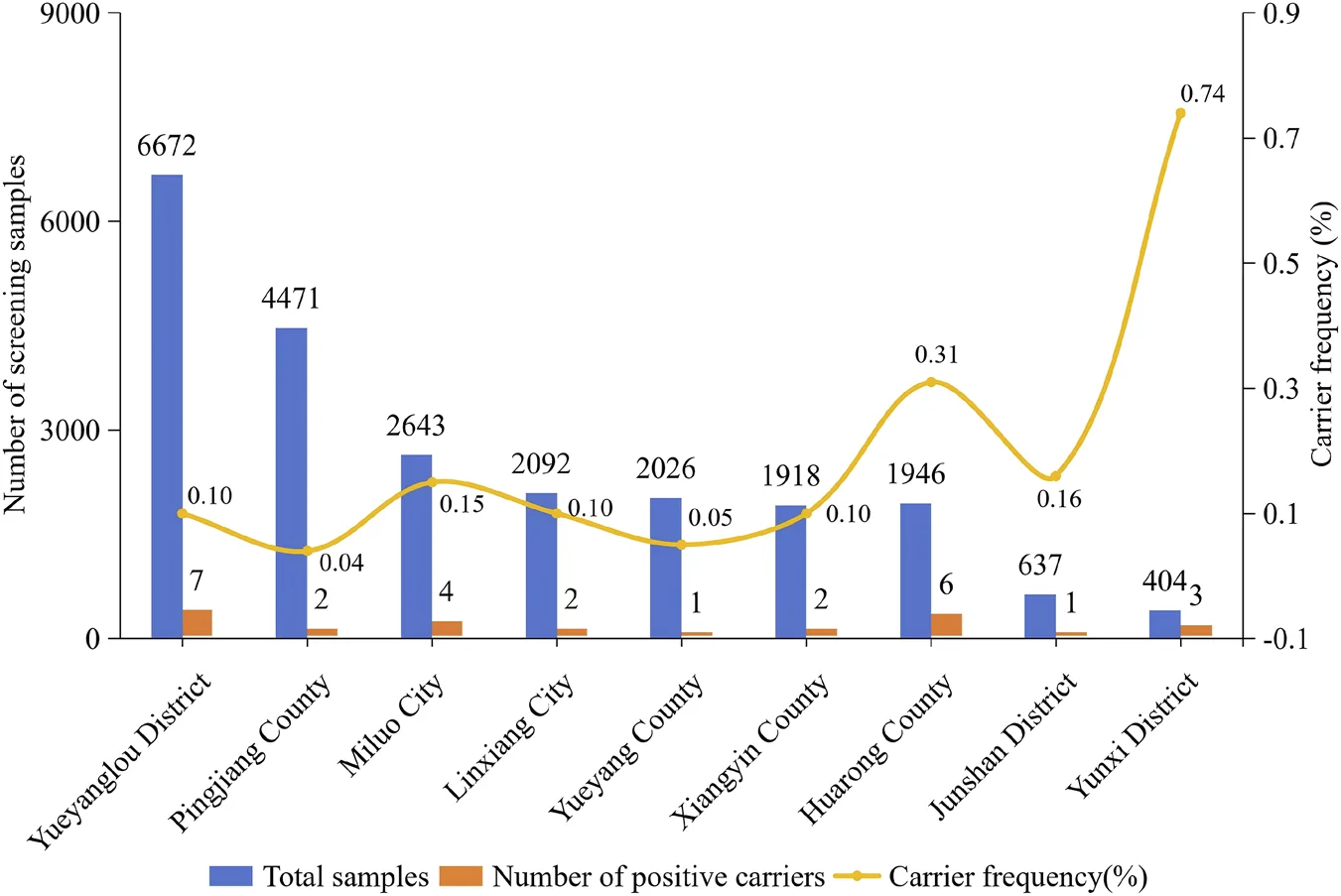
The left y-axis: number of screening samples; the right y-axis: carrier frequency; the x-axis: district. Bars depict absolute counts (blue: total samples; orange: positive carriers). The line graph represents the carrier frequency (%). Yuyanglou District had the highest sample volume; Yunxi District had the highest carrier rate.

Figure 3 and Table 2 present the DMD carrier rates among female screening samples from Yueyang City. Yunxi District showed the highest rate (0.74%), followed by Huarong County (0.31%). Junshan District has the third-highest rate at 0.16%. Lower rates were observed in Yueyanglou District (0.11%), Xiangyin County (0.10%), Linxiang City (0.10%), Yueyang County (0.05%), and Pingjiang County (0.05%).

**TABLE 2:** Carrier rate of *DMD* variants across districts and counties in Yueyang City.

Inspection area	Submitted samples	Positive samples	Carrier rate (%)
Yunxi district	404	3	0.74
Huarong County	1946	6	0.31
Miluo City	2,643	4	0.15
Junshan district	637	1	0.16
Yueyanglou district	6,672	7	0.11
Xiangyin County	1918	2	0.10
Linxiang City	2092	2	0.10
Yueyang County	2026	1	0.05
Pingjiang County	4,471	2	0.05

Analysis of the geographical distribution of *DMD* mutations (Table 3) revealed distinct patterns in regions with high positive sample counts.

1. SNVs and indels exhibited low detection rates with random genomic distributions.
2. CNVs exhibited significantly higher detection rates than SNVs/indels (71.43% vs.28.57%), with predominant clustering in exons 45–71, a region critical for dystrophin stability. Geographic heterogeneity was evident: deletion hotspots localized to exons 45–55 (Yueyanglou District, frequency: 51.8%), exons 3–7 (Linxiang City), and exons 30–44 (Junshan District).

**TABLE 3:** Genetic and demographic characteristics of DMD carrier-positive samples.

Inspection area	Variant position	Number of variant	Total number
Yueyanglou district	c.10454del	1	7
	c.1615C>T	1	
	c.4300A>T	1	
	E46-52del	1	
	E48-49del	1	
	E48del	1	
	E51-52del	1	
Huarong County	c.1812 + 1G>A	1	6
	c.3502G>T	1	
	E45-55del	1	
	E46-54del	1	
	E50-59del	1	
	E54-62del	1	
Miluo City	E45del	1	4
	E45-49dup	1	
	E48-49del	1	
	E51-60dup	1	
Yunxi district	c.5287C>T	1	3
	E48del	1	
	E68-71del	1	
Linxiang City	c.9100C>T	1	2
	E48-53del	1	
Xiangyin County	E3-7del	1	2
	E51-52del	1	
Pingjiang County	c.10231dup	1	2
	E48-55del	1	
Yueyang County	E56-62del	1	1
Junshan district	E30-44del	1	1

E, exon. Del, deletion. Dup, duplication.

### 3.3 *DMD* gene mutation analysis

In this study, we analyzed 28 DMD-positive samples, corresponding to 25 distinct P/LP variants (Table 4). Of these, 8 (28.57%) harbored SNV/indels variants and 20 (71.43%) harbored CNVs. By mutation type, the SNV/indels variants comprised 5 nonsense (17.86%), 2 frameshift (7.14%), and 1 splice site (3.57%) mutations. Among the nonsense mutations, c.3502G>T (p.E1168*) was previously unreported in major public databases. The CNVs consisted of 18 exon deletions (64.29%) and 2 exon duplications (7.14%).

**TABLE 4:** Carrier identification of P/LP variants in *DMD* gene.

Variant type		Variant position	Number	Proportion
SNV/indels	Nonsense variant	c.1615C>T (p.R539*)	1	17.86% (5/28)
		c.3502G>T (p.E1168*)	1	
		c.4300A>T (p.K1434*)	1	
		c.5287C>T (p.R1763*)	1	
		c.9100C>T (p.R3034*)	1	
	Frameshift variant	c.10231dup (p.T3411Nfs*22)	1	7.14% (2/28)
		c.10454del (p.L3485Rfs*11)	1	
	Splice region variant	c.1812 + 1G>A	1	3.57% (1/28)
CNV	Exon deletion	E3-7del	1	64.29% (18/28)
		E30-44del	1	
		E45del	1	
		E45-55del	1	
		E46-52del	1	
		E46-54del	1	
		E48-49del	2	
		E48-53del	1	
		E48-55del	1	
		E48del	2	
		E50-59del	1	
		E51-E52del	2	
		E54-62del	1	
		E56-62del	1	
		E68-71del	1	
	Exon duplication	E45-49dup	1	7.14% (2/28)
		E51-60dup	1	

E, exon. Del, deletion. Dup, duplication. CNV, copy number variant; indel, insertion/deletion. SNV, single nucleotide variant.

Pathogenicity was assessed for these variants (Table 5). Of the 28 positive samples, 15 variants were pathogenic (53.57%) and 13 were likely pathogenic (46.43%). By variant type, among the 8 SNV/indels variants, 4 were pathogenic (50.00% of SNV/indels, 14.29% of all variants) and 4 were likely pathogenic (50.00% of SNV/indels, 14.29% of all variants). Among the 20 CNVs, 11 were pathogenic (55.0% of CNVs, 39.29% of all variants) and 9 were likely pathogenic (45.00% of CNVs, 32.14% of all variants).

**TABLE 5:** Distribution of *DMD* variants across pathogenicity categories.

Variant type		Number of carriers	Proportion
SNVs/indels	P	4	14.29%
	LP	4	14.29%
CNV	P	11	39.29%
	LP	9	32.14%

CNV, copy number variant. Indel, insertion/deletion. SNV, single nucleotide variant.

### 3.4 DMD SNV/indel analysis

**FIGURE 4:**
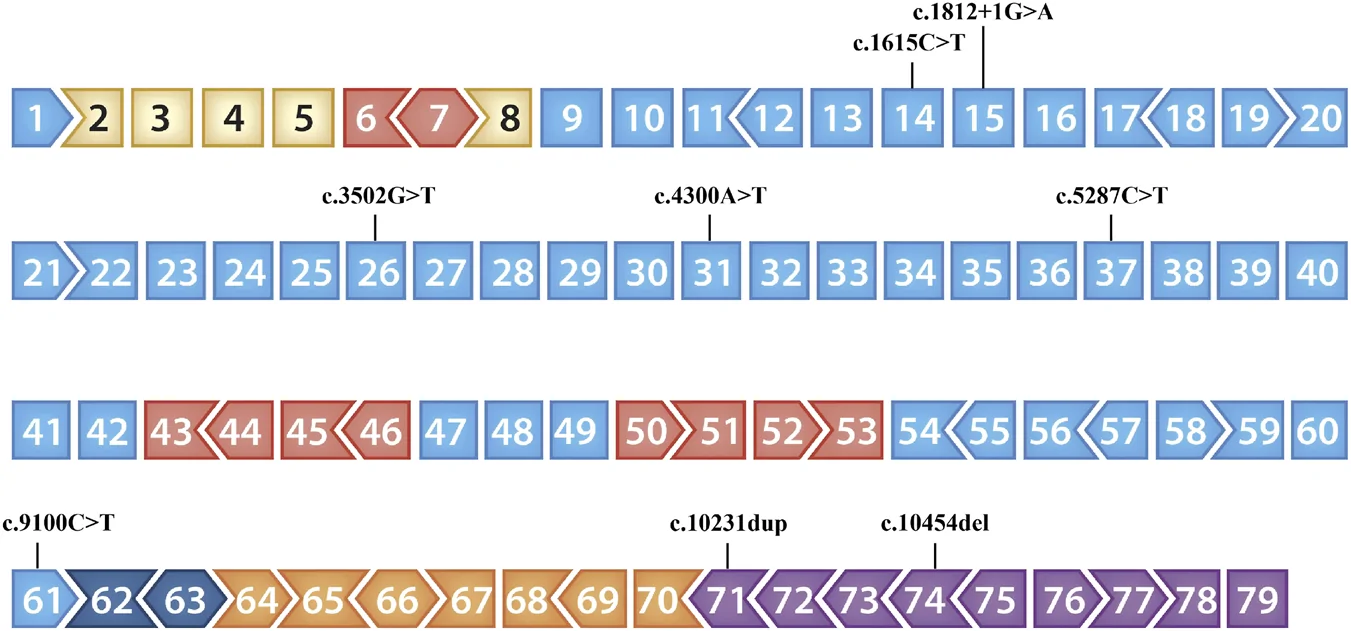
Distribution of single-nucleotide variants (SNVs) in the DMD gene among positive carriers. The schematic of DMD exons is adapted from (Min et al., 2019).

Using Mutalyzer 3 (https://www.mutalyzer.nl/), we mapped the exon coordinates of SNV/indels variants in the *DMD* gene. Nonsense mutations were detected in exons 14, 26, 31, and 37; frameshift mutations in exons 71 and 74; and splice site mutations affecting exon 15 (Table 6; Figure 4).

**TABLE 6:** Detection of SNVs/indels in *DMD* gene.

Sample ID	Maternal age (years)	Gestational age (weeks)	Sample location	Variant type	Exon location	Nucleotide change	Amino acid change
3	32	10^+6^	Huarong County	Nonsense variant	E26	c.3502G>T	p.E1168*
5	36	16^+1^	Huarong County	Splice region variant	E15	c.1812 + 1G>A	—
6	29	7	Yueyanglou district	Nonsense variant	E31	c.4300A>T	p.K1434*
8	27	8^+3^	Pingjiang County	Frameshift variant	E71	c.10231dup	p.T3411Nfs*22
14	36	7^+1^	Yueyanglou district	Frameshift variant	E74	c.10454del	p.L3485Rfs*11
21	30	4^+6^	Yueyanglou district	Nonsense variant	E14	c.1615C>T	p.R539*
25	21	0	Linxiang City	Nonsense variant	E61	c.9100C>T	p.R3034*
28	31	12^+3^	Yunxi district	Nonsense variant	E37	c.5287C>T	p.R1763*

E, exon. Del, deletion. Dup, duplication.

### 3.5 *DMD* exon deletion/duplication analysis

Analysis of 18 exon deletion cases revealed that single-exon deletions constituted 16.66% (3/18), with exon 48 deletions (2/18, 11.11%) being more frequent than exon 45 deletions (1/18, 5.55%). In contrast, multi-exon deletions (≥2 exons) accounted for 83.33% (15/18). The largest contiguous deletion spanned exons 30–44 (15 exons; 25.8 kb) in one sample, and 72.22% (13/18) of deletions clustered in the exon 45–55 region.

**FIGURE 5:**
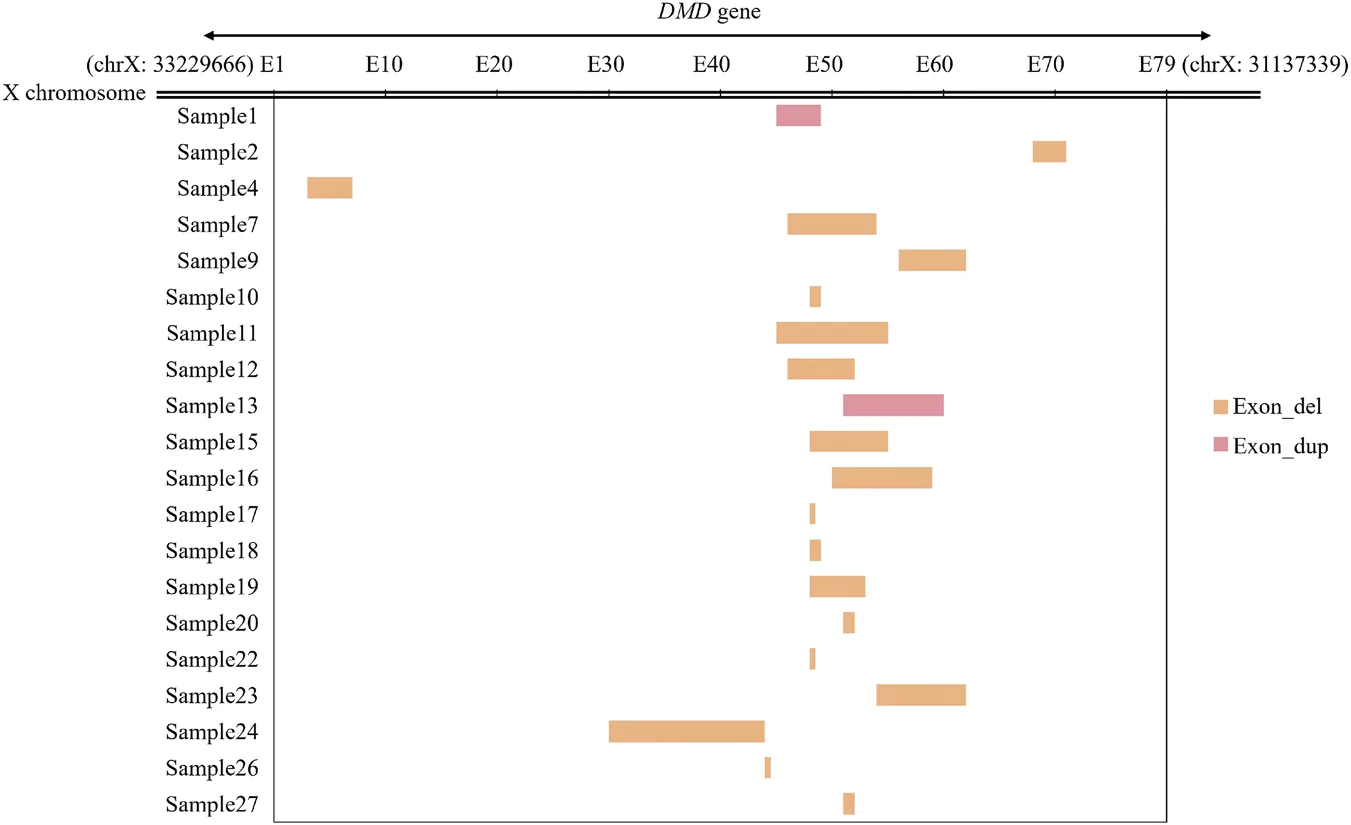
The distribution of variants in positive cases involving exon duplications and deletions. Orange represents deletions, whereas pink represents duplications. The hotspots clustered in the exon 45–55 region.

Two exon duplication cases were identified, both involving multi-exon events: one at exons 45–49 (5 exons) and another at exons 51–60 (10 exons). The duplicated regions partially overlapped the exons 45–55 deletion hotspot. Deletion and duplication distributions are summarized in Figure 5.

**FIGURE 6:**
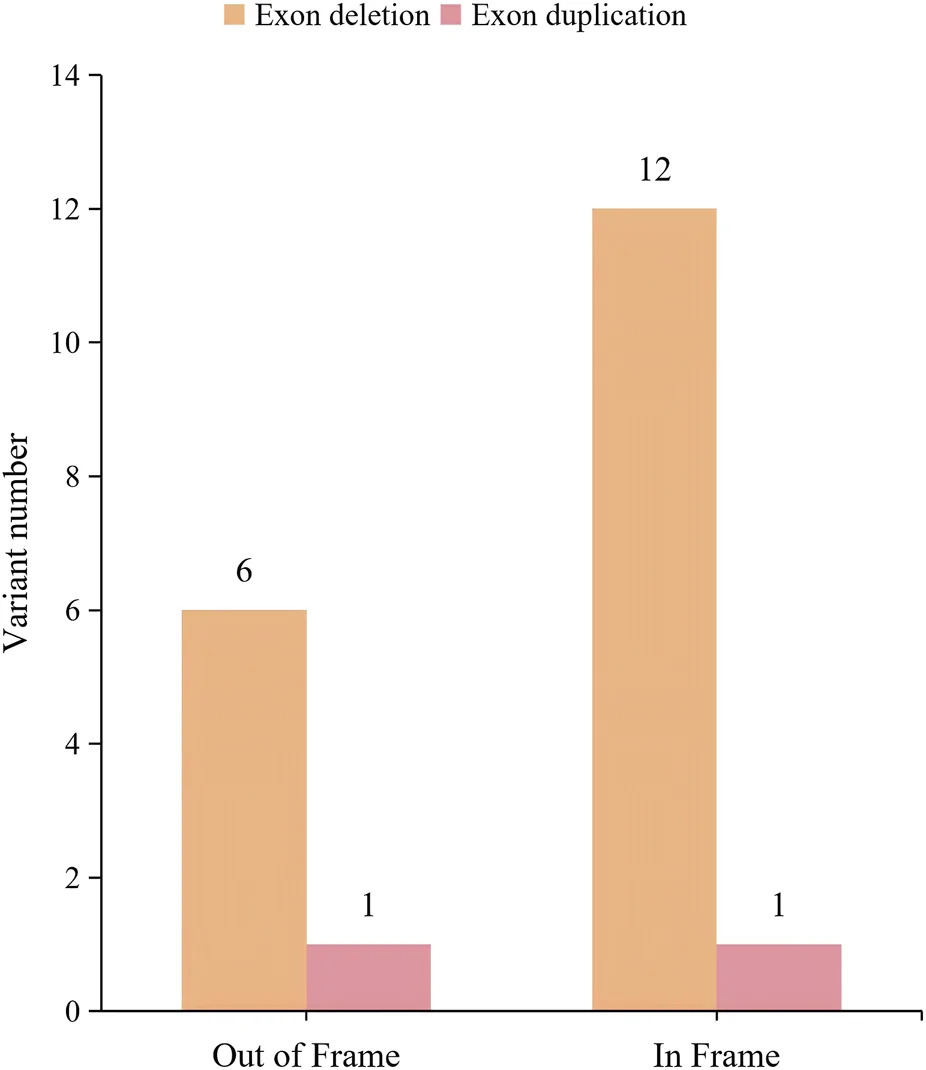
Analysis of exon deletion and duplication variants based on the reading frame principle. Orange represents deletions; pink represents duplications.

Using the Leiden Open Variation Database (LOVD) for Duchenne Muscular Dystrophy (http://www.dmd.nl/), we assessed reading-frame rule compliance in 28 dystrophin gene mutations from DMD carriers. Of 18 exon deletion cases, 6 (33.33%) violated the reading-frame rule (out-of-frame), while 12 (66.66%) preserved an open reading frame (in-frame). Among two exon duplication cases, one exhibited an out-of-frame and one an in-frame configuration (Figure 6).

### 3.6 Detection of VUS in the *DMD* gene

Among the 81 VUSs, corresponding to 51 distinct variant types, 64 (79.01%) were classified as SNVs or indels, and 17 (20.99%) as CNVs. Missense mutations constituted the majority of VUS (54/81, 66.67%), significantly exceeding splice site mutations (10/81, 12.35%) (Table 7). 11 variants - c.3502G>T (p.E1168*), c.1096G>A (p.G366R), c.118C>T (p.L40F), c.5044_5045delinsTT (p.E1682L), c.5188A>G (p.K1730E), c.5190G>T (p.K1730N), c.7918G>A (p.A2640T), c.7981A>T (p.M2661L), c.804A>C (p.L268F), c.8658G>T (p.Q2886H), c.9084 + 2T>C, and c.965_967dup (p.L322_E323insV) - were absent from all queried databases, indicating that these variants have not been previously reported. For CNV subtypes, exon duplications (14/81, 17.28%) were more frequent than exon deletions (3/81, 3.70%).

**TABLE 7:** Characteristics of VUS in *DMD* gene.

Variant type		Variant position	Nucleotide change	Number	Proportion
SNV/indels	Splice region variant	c.10224-1G>T	—	2	12.35% (10/81)
		c.10224–6_10230del	—	1	
		c.7098 + 2T>C	—	1	
		c.7661–11T>C	—	3	
		c.9084 + 2T>C	—	2	
		c.264 + 5G>C	—	1	
	Missense variant	c.1096G>A	p.G366R	1	66.67% (54/81)
		c.118C>T	p.L40F	1	
		c.1320A>T	p.E440D	1	
		c.167G>A	p.G56D	2	
		c.2600A>C	p.K867T	1	
		c.2794C>G	p.L932V	1	
		c.3083G>A	p.R1028H	1	
		c.3194C>T	p.A1065V	1	
		c.4020C>G	p.I1340M	4	
		c.4030C>T	p.L1344F	7	
		c.4295A>C	p.Q1432P	1	
		c.4296G>C	p.Q1432H	4	
		c.4604C>T	p.T1535M	1	
		c.4798G>A	p.V1600I	1	
		c.5044_5045delinsTT	p.E1682L	1	
		c.5153A>G	p.K1718R	1	
		c.5188A>G	p.K1730E	1	
		c.5190G>T	p.K1730N	1	
		c.5203C>T	p.R1735C	1	
		c.5701G>T	p.A1901S	1	
		c.6152G>A	p.R2051Q	4	
		c.772C>T	p.P258S	1	
		c.7892G>A	p.R2631H	2	
		c.7918G>A	p.A2640T	1	
		c.7981A>T	p.M2661L	2	
		c.804A>C	p.L268F	1	
		c.8626C>A	p.Q2876K	2	
		c.8658G>T	p.Q2886H	1	
		c.941G>A	p.R314Q	2	
		c.9437A>C	p.D3146A	1	
		c.9438T>G	p.D3146E	3	
		c.965_967dup	p.L322_E323insV	1	
CNV	Exon deletion	E17-28del	—	1	3.70% (3/81)
		E49del	—	1	
		E54-59del	—	1	
	Exon duplication	E1-2dup	—	2	17.28% (14/81)
		E1-4dup	—	1	
		E13dup	—	3	
		E1dup	—	2	
		E38-41dup	—	1	
		E41dup	—	1	
		E48-49dup	—	1	
		E62-79dup	—	1	
		E63-79dup	—	1	
		E79dup	—	1	

E, exon. CNV, copy number variant. Indels, insertion/deletion. SNV, single nucleotide variant.

### 3.7 Follow-up results

**FIGURE 7:**
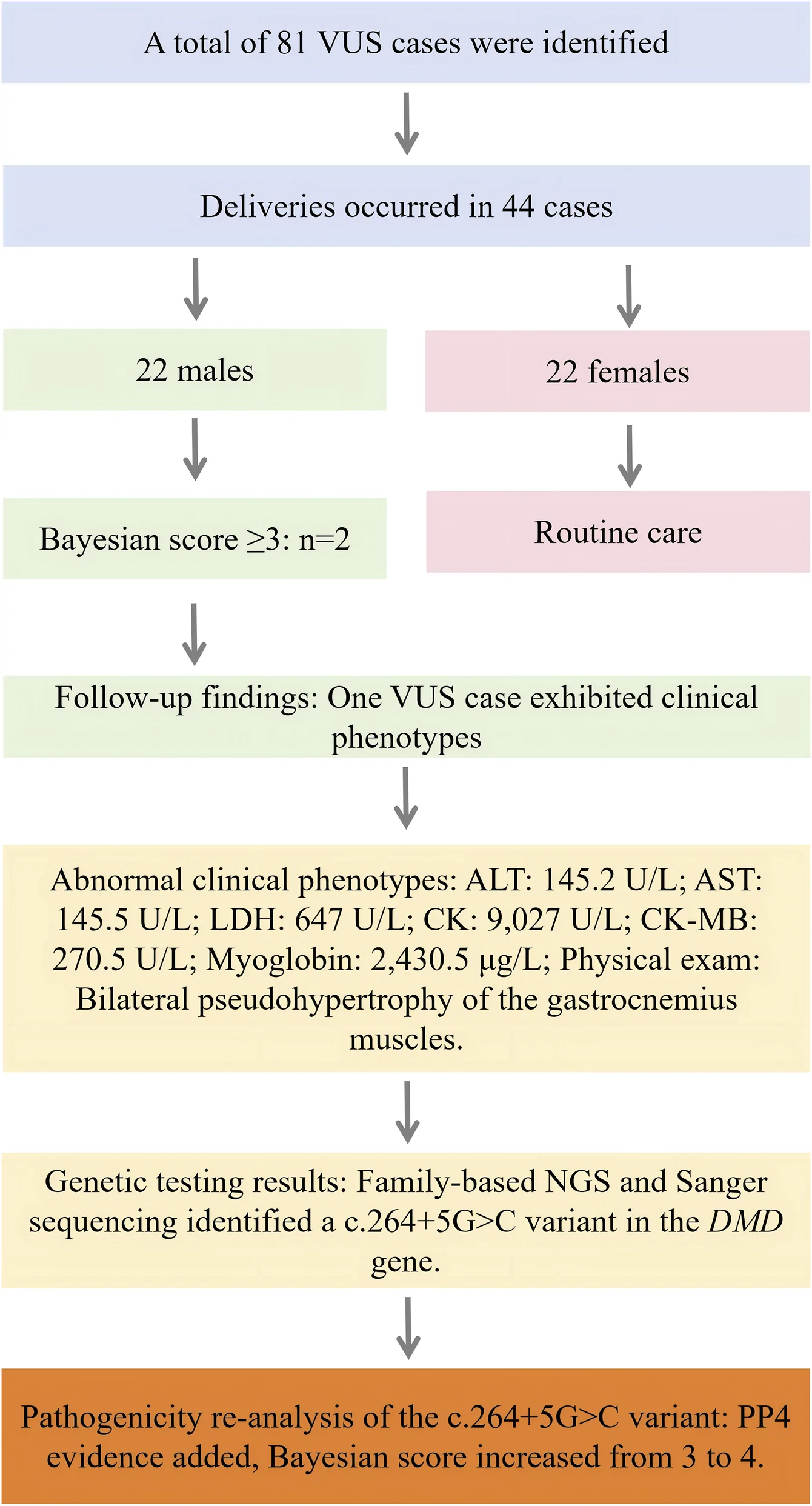
ALT: Alanine aminotransferase; AST: Aspartate aminotransferase; LDH: Lactate dehydrogenase; CK: Creatine kinase; CK-MB: CK-MB isoenzyme. NGS: Next-Generation Sequencing.

**FIGURE 8:**
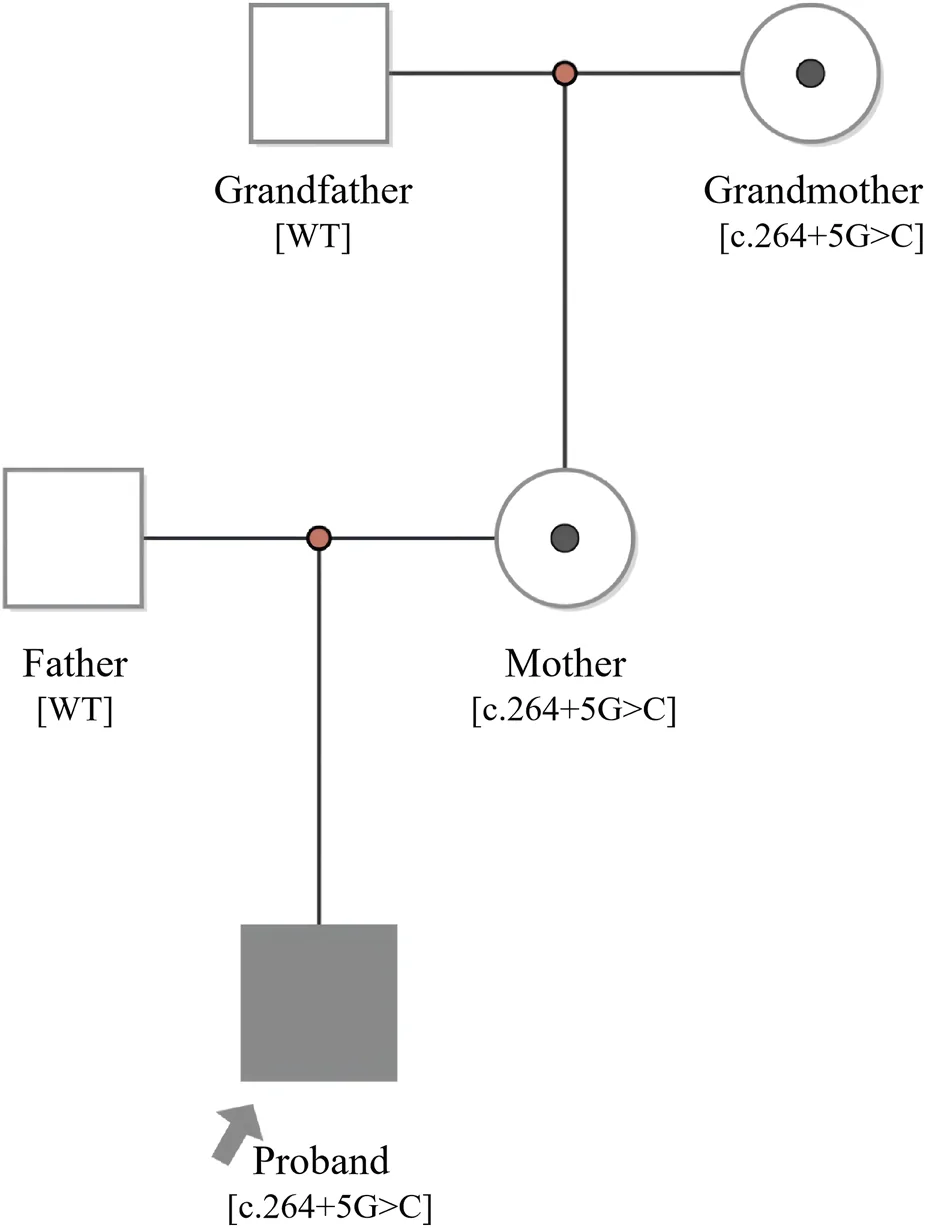
Squares denote males; circles denote females. A circle with a central dot indicates a female carrier. A filled square indicates the proband (affected male). WT: wild type.

Of the 81 VUSs identified, 44 carriers had delivered live-born infants, and 37 were either non-pregnant or had not yet delivered. Among the live-born infants, there were 22 males and 22 females. From the male infants, we selected two with a Bayesian score ≥3 for enhanced monitoring. Ultimately, one infant carrying the VUS c.264 + 5G>C presented with a clinical phenotype; the detailed manifestations are summarized in Figure 7. Laboratory testing showed markedly elevated serum CK (9,027 U/L), CK-MB (270.5 U/L), myoglobin (2,430.5 μg/L), LDH (647 U/L), and transaminases (ALT 145.2 U/L, AST 145.5 U/L). Physical examination additionally revealed bilateral calf pseudohypertrophy. These findings constitute a highly specific phenotype for DMD in a hemizygous male, supporting the application of PP4 at moderate strength according to the ACMG/AMP and ClinGen Sequence Variant Interpretation guidelines. Accordingly, the Bayesian score for the c.264 + 5G>C variant was upgraded from 3 to 4. Figure 8 shows the family pedigree illustrating segregation of the c.264 + 5G>C variant in the *DMD* gene. In addition to the VUS follow-up described above, the outcomes of the 28 P/LP carriers are presented in Table 8.

**TABLE 8:** Follow-up results of DMD carrier-positive samples.

Sample ID	Variant position	ACMG pathogenicity	Family history	Prenatal diagnosis	Prenatal phenotype	Postartum phenotype
1	E45-49dup	LP	No	Not pregnant	—	—
2	E68-71del	P	No	Not detected	No	No
3	c.3502G>T (p.E1168*)	LP	No	Not detected	No	No
4	E3-7del	P	Her cousin is a patient with DMD.	Not detected	No	No
5	c.1812 + 1G>A	LP	Her brother and uncle are carriers of DMD.	c.1812 + 1G>A	No	No
6	c.4300A>T (p.K1434*)	LP	Loss to follow-up			
7	E46-54del	LP	No	Not pregnant	—	—
8	c.10231dup (p.T3411Nfs*22)	LP	No	Not pregnant	—	—
9	E56-62del	P	No	E56-62del	No	No
10	E48-49del	P	No	E48-49del	No	No
11	E45-55del	P	No	Not detected	No	No
12	E46-52del	P	No	Not detected	No	No
13	E51-60dup	LP	Loss follow-up			
14	c.10454del (p.L3485Rfs*11)	P	No	Not detected	No	No
15	E48-55del	P	No	E48-55del	No	No
16	E50-59del	LP	No	Not pregnant	—	—
17	E48del	P	No	Not detected	No	No
18	E48-49del	P	Her brother died at age 20. The initial symptom was difficulty ascending stairs, followed by difficulty walking	c.9238-9241del	No	Unborn
19	E48-53del	LP	No	Not pregnant	—	—
20	E51-52del	LP	No	E51-52del	No	Termination of pregnancy
21	c.1615C>T (p.R539*)	P	Have given birth to a child with DMD.	c.1615C>T	No	Unborn
22	E48del	P	No	Not detected	Bright spot in the left ventricle	Unborn
23	E54-62del	P	No	E54-62del	No	Unborn
24	E30-44del	LP	No	Not pregnant	—	—
25	c.9100C>T (p.R3034*)	P	No	Not pregnant	—	—
26	E45del	LP	No	E45del	No	Unborn
27	E51-52del	LP	No	No	—	—
28	c.5287C>T (p.R1763*)	P	No	c.5287C>T	No	Unborn

ACMG, American College of Medical Genetics; LP, likely pathogenic. P, pathogenic.

## 4 Discussion

This population-based study was designed to determine the carrier frequency and mutation spectrum of DMD among women of childbearing age and early pregnancy in Yueyang City, China, with the aim of providing evidence for regional genetic counseling and prevention strategies. Our analysis of 25,611 participants yielded three principal findings. First, the overall carrier rate of P/LP variants was 0.11%, with CNVs accounting for 71.43% of all mutations. Second, we observed marked geographical heterogeneity, with the carrier rate in Yunxi District reaching 0.74%. Third, and most importantly, we identified 81 VUS cases, including one prenatally detected VUS that correlated with a postnatal clinical phenotype, underscoring the previously underappreciated clinical relevance of VUS in DMD carrier screening.

Comparative analysis of DMD screening methods (Table 9) revealed that the biochemical CK-MM assay generated an elevated initial screen-positive rate. Three large-scale studies (n = 50,000; 36,781; 13,110) reported initial screen-positive rates of 1.26%, 0.11%, and 0.84%, respectively (Chien et al., 2022; Tavakoli et al., 2023; Jia et al., 2023). However, after applying confirmatory tests-including repeat CK-MM testing or genetic validation via NGS/MLPA-screen-positive rates declined substantially to below 0.05% in all cohorts, likely due to the exclusion of false positives caused by non-specific neonatal muscle damage. Although CK-MM screening is operationally convenient and cost-effective, its high false-positive rate necessitates secondary genetic confirmation for definitive diagnosis. Research indicates that neonatal CK-MM levels are significantly influenced by factors including postnatal age, gestational age, birth weight, and specimen collection timing (Maloney et al., 2023). Gender, ethnicity, and seasonal temperature variations also contribute to CK-MM variability. Therefore, combining CK-MM screening with confirmatory genetic testing is essential for accuracy. MLPA is the gold standard for *DMD* mutation detection (Hua et al., 2023). In a cohort of 12,362 screened neonates, MLPA identified a carrier rate of 0.07% (9/12,362) (Cohen et al., 2022). However, MLPA’s operational complexity and cost limit its utility in large-scale screening (Nallamilli et al., 2021). Alternative strategies using NGS for initial screening followed by MLPA confirmation of positive cases demonstrated a carrier rate of 0.10% (82/85,737) (Singer et al., 2023), aligning with our study’s findings. When used alone, NGS may yield false positives due to inadequate probe coverage, bioinformatic errors in variant calling, low DNA quality or sequencing depth (Huang et al., 2022). This study optimized the bioinformatics analysis workflow and designed encrypted probes targeting the 79 exonic regions of the *DMD* gene, supplemented by anchoring probes in flanking intronic regions to precisely detect exon-level deletions/duplications. This design reduced NGS false positives by enhancing hybridization specificity. Consequently, NGS-based initial screening outperformed stand-alone NGS without intronic anchors. Therefore, a combined strategy using optimized NGS followed by MLPA confirmation is optimal for large-scale DMD screening.

**TABLE 9:** The application of different detection methods in DMD screening.

No.	Sample type	Number	Detection method	Verification method	Screening positive rate (%)	Carriage rate (%)	Study
1	Newborn	50,000	CK-MM	CK-MM	1.26	0.03	Chien et al. (2022)
2	Newborn	36,781	CK-MM	NGS	0.11	0.01	Tavakoli et al. (2023)
3	Newborn	13,110	CK-MM	MLPA	0.84	0.05	Jia et al. (2023)
4	Adult	91,637	NGS	—	0.15	—	Souter et al. (2023)
5	Adult	85,737	NGS	MLPA	0.53	0.10	Singer et al. (2023)
6	Adult	12,362	MLPA	—	0.07	0.07	Cohen et al. (2022)
7	Adult	25,611	NGS	MLPA	0.17	0.11	Our study

CK-MM, creatine kinase muscle-type; NGS, next-generation sequencing; MLPA, Multiplex ligation-dependent probe amplification.

The practice guidelines for genetic testing in DMD (2020) indicate that the pathogenic variant spectrum of the DMD gene exhibits the following characteristics: exon deletions or duplications are the predominant variant types, accounting for 78% of cases (68% deletions, 10% duplications) (Fratter et al., 2020). The remaining 22% comprise SNVs and indels, among which nonsense mutations and frameshift variants account for 9% and 7%, respectively, while splice-site variants represent 5.6%. This distribution is largely consistent with our findings. A 2021 study utilizing a DMD-specific gene panel reported that 65% of deletions clustered in distal hotspots (exons 45–55), whereas 17% occurred in proximal regions (exons 1–20); the remaining deletions were evenly distributed in intermediate regions (exons 18–45) (Arun et al., 2021). In our cohort, however, 72.22% (13/18) of deletions localized to the exon 45–55 region, with only ∼5% in proximal and intermediate regions combined. This discrepancy likely reflects differences in sample sources and probe design methodologies. Point mutations, in contrast, were randomly distributed across both distal and proximal hotspots-a pattern aligned with our results. Notably, 12 variants (1 LP and 11 VUSs) were previously unreported, including c.3502G>T (p.E1168*; LP), c.1096G>A (p.G366R), c.118C>T (p.L40F), c.5044_5045delinsTT (p.E1682L), c.5188A>G (p.K1730E), c.5190G>T (p.K1730N), c.7918G>A (p.A2640T), c.7981A>T (p.M2661L), c.804A>C (p.L268F), c.8658G>T (p.Q2886H), c.9084 + 2T>C, c.965_967dup (p.L322_E323insV), thereby expanding the known mutational spectrum of DMD.

In previous studies, the impact of *DMD* gene mutations has primarily been associated with whether these mutations disrupt the downstream reading frame. When out-of-frame mutations occur, they disrupt the open reading frame of the *DMD* gene, causing disruption and leading to dystrophin deficiency, which results in severe DMD phenotypes. In contrast, in-frame mutations preserve the reading frame, allowing the production of partially functional dystrophin protein, which results in the milder BMD phenotype (Zhou et al., 2021; Sun et al., 2020). This open reading frame rule holds true for approximately 90% of cases (Fortunato et al., 2021), with notable exceptions where in-frame mutations are associated with severe DMD phenotypes. However, studies have indicated that intragenic deletion patterns may result in a mixed phenotype of DMD and BMD (Sheikh and Yokota, 2020). The deletions associated with DMD include exons 3–13, 3–29, 3–37, 6–42, 9–55, as well as exons 25 and 31–34 (Cohen et al., 2022; Anwar et al., 2021). The functionality of the dystrophin protein encoded by the DMD gene is significantly impaired by large deletions in its central rod domain. These mutations typically cause DMD rather than BMD (Sagi-Dain et al., 2024), as they can disrupt normal pre-mRNA processing and subsequent protein translation. This severe outcome arises because such deletions frequently impact critical functional domains of dystrophin, thereby abolishing its normal function and leading to the severe clinical manifestations characteristic of DMD (Mathur et al., 2025). The c.372delG mutation in exon 6, as well as splice site mutations, have been demonstrated to result in the incorporation of a pseudo-exon and the creation of a premature stop codon. Such mutations are linked to severe forms of DMD due to the production of truncated, dysfunctional dystrophin that undergoes rapid degradation (Zhang et al., 2019). In summary, differentiating DMD from BMD extends beyond the reading frame rule. A comprehensive assessment must account for the specific genomic locations of mutations (deletions, duplications, or point mutations) and their consequent effects on dystrophin protein function, especially within its critical structural domains.

Female carriers of X-linked recessive disorders, such as those with pathogenic variants in the DMD gene, are not invariably asymptomatic. While the presence of a normal allele on the second X chromosome can provide compensation, the random nature of X-chromosome inactivation (XCI) means that some carriers may experience disease manifestations. This is reflected in the terminology used in a 2021 study, which classified such individuals as ‘DMD carriers’ (Basta and Pandya, 2023; Apkon et al., 2021). Cardiac involvement is prevalent among female carriers of DMD. A study by Mah et al. (Mah et al., 2020) found that 49% of these carriers showed evidence of myocardial fibrosis on cardiac MRI, which was characterized by significant left ventricular dilation and impaired ejection fraction. Beyond cardiac manifestations, this population may also experience skeletal muscle weakness and are at potential risk for developing cardiomyopathy (Bourke et al., 2022). Additionally, a wider range of complications has been documented, including arrhythmias, rhabdomyolysis, respiratory dysfunction, airway difficulties, and coagulation disorders, as summarized by Cullom et al. (Cullom et al., 2022). The reported prevalence of symptomatic manifestations among female DMD carriers varies considerably across studies. For instance, research from 2019 indicated that approximately 5% of carriers exhibited clinical symptoms (Zhong et al., 2019). In contrast, a more recent large-scale investigation in 2023 found a substantially higher proportion, with 25.7% of carriers being symptomatic (Liu et al., 2023). This pronounced heterogeneity in clinical presentation is often attributed to differences in XCI, where a skewed pattern favoring the inactivation of the X chromosome carrying the normal DMD allele can lead to more severe manifestations. Recent studies (Politano, 2025) on DMD/BMD suggest that symptomatic female carriers often exhibit a skewed XCI pattern, frequently with preferential inactivation of the X chromosome carrying the normal allele. However, it is important to note that a clear correlation between XCI skewing and symptomatic status is not universally observed, as some asymptomatic carriers also show skewed inactivation, while some symptomatic carriers display a random pattern. Furthermore, tissue-specific mosaicism in XCI patterns is recognized, meaning the degree of skewing can vary between different tissues (e.g., blood, muscle, heart). This mosaicism may significantly modify the severity of clinical expression in carriers (Pietro et al., 2025). From a clinical and reproductive perspective, female carriers of a DMD mutation have a 50% chance of passing the pathogenic variant to their offspring in each pregnancy, resulting in a 25% chance of having an affected son (Pickart et al., 2025). Therefore, offering carrier screening before or early in pregnancy is crucial. This allows identified carriers to undergo genetic counseling, where they can fully understand their personal recurrence risks and reproductive options (Han et al., 2020).

Carrier screening is typically offered to couples of childbearing age or during early pregnancy. In this context, and in the absence of phenotypic and familial information, VUS are frequently underinterpreted. Consequently, genetic screening programs should encompass not only P and LP variants but also VUS, which warrant careful reporting and follow-up. The pathogenicity of these VUS can often be clarified through additional functional assays or the collection of supplemental clinical and familial data.

A detailed family history provides critical evidence for the interpretation of VUS pathogenicity (Hanson et al., 2023). Furthermore, cascade testing of family members (familial segregation analysis) is instrumental in determining whether a variant is inherited or *de novo* (Rehm et al., 2023). When a family history of hereditary disease is associated with a specific gene, multiple lines of evidence can be utilized. The observation of co-segregation of the variant with the disease within the family supports the application of the PP1 evidence criterion, which contributes to the accumulation of evidence favoring pathogenicity (Biesecker et al., 2024). Additionally, the PS2 evidence criterion is critical: identification of a confirmed *de novo* variant in a patient with a consistent disease phenotype can strongly support pathogenicity (Zhang et al., 2024). Moreover, in the context of prenatal carrier screening, if a fetus carrying a VUS subsequently exhibits a clinical phenotype consistent with the suspected disease after birth, this provides PP4 evidence (phenotype-specific evidence) that further strengthens the likelihood of pathogenicity (Biesecker et al., 2024). Consistent with this principle, in our cohort, the DMD VUS c.264 + 5G>C (Bayesian score 3) was re-evaluated after postnatal follow-up: the male infant demonstrated markedly elevated CK (9,027 U/L), CK-MB, myoglobin, LDH, and transaminases of muscle origin, along with bilateral calf pseudohypertrophy. These highly specific phenotypic findings enabled the application of PP4 at moderate strength, increasing the Bayesian score from 3 to 4. Collectively, leveraging family history to obtain co-segregation data (PP1), confirming *de novo* status (PS2), and incorporating postnatal phenotypic evidence (PP4) enables a more comprehensive and accurate assessment of VUS pathogenicity, improving classification accuracy and reducing the risk of missed diagnoses (Riggs et al., 2021).

However, the application of PP1 and PS2 in DMD requires careful consideration of several caveats specific to X-linked inheritance. First, because DMD is an X-linked recessive disorder, segregation analysis in female carriers is often confounded by skewed X-inactivation, which can result in a broad spectrum of clinical presentations-from asymptomatic to severely affected-thereby weakening the reliability of co-segregation patterns for PP1 (Politano, 2025). Second, germline mosaicism occurs at a substantially high frequency in DMD, meaning that a variant appearing *de novo* in a proband may actually be present in the germ cells of an apparently unaffected mother. This phenomenon complicates the confirmation of true *de novo* status (PS2) and can lead to misclassification of recurrence risk (Verebi et al., 2025). These limitations underscore that PP1 and PS2 should be applied with caution when assessing DMD variants, and whenever possible, complementary evidence (such as mRNA splicing analysis) should be integrated to refine VUS classification.

Given the inherent uncertainty of VUS and the practical constraints of population-scale carrier screening, we propose a stepwise, phenotype-driven management protocol for *DMD* VUS (Bayesian score ≥3; see Methods for scoring) detected in female carriers. This protocol was approved by the Institutional Ethics Committee, and the informed consent explicitly stated that VUS would not be returned to asymptomatic carriers (Mighton et al., 2026). Under this non-disclosure strategy, the VUS is internally flagged; only the carrier’s male offspring are enrolled in active surveillance (Step 1: serial CK/CK-MB measurements at birth, 6 months, 18 months, and annually, along with calf examination and motor milestone assessments).

Importantly, the four clinical management elements recommended for VUS management - detailed family history, maternal CK assessment, segregation analysis, and RNA studies - are not performed at the time of maternal carrier identification. Instead, they are triggered only when the male offspring exhibits phenotypic abnormalities (e.g., persistent CK elevation, calf pseudohypertrophy, or motor delay). At that point, comprehensive genetic diagnosis is initiated (Step 2: full *DMD* sequencing plus CNV analysis on the proband, with parental validation). During this diagnostic workup, the following evaluations are performed concurrently:

Detailed family history: A three-generation pedigree is obtained from the mother, with specific attention to any male relatives with elevated CK, muscle weakness, or unexplained cardiomyopathy. This contextualizes the VUS without requiring prior disclosure of carrier status.

Maternal serum CK: Measured alongside the proband’s diagnostic blood draw; even mild elevations can indicate subclinical myopathy in the mother and support variant reclassification.

Segregation analysis: Trio-based sequencing (mother, father, proband) is performed to determine whether the VUS is inherited from the mother (consistent with X-linked transmission) or arises de novoin the affected son. If additional affected male relatives are available, extended segregation is pursued to evaluate genotype-phenotype co-segregation.

RNA studies: If the VUS remains classified as non-LP/P after segregation and bioinformatic re-analysis, targeted RNA studies (Step 3) - preferably from muscle biopsy or blood-based RNA sequencing - are recommended for either the symptomatic proband or the mother (if she has elevated CK or clinical symptoms). These studies can detect aberrant splicing and potentially upgrade the VUS to likely pathogenic.

Concurrent longitudinal re-analysis of the VUS is performed every 1–2 years. This tiered approach leverages the observation that persistently normal CK and motor findings through early childhood strongly argue against pathogenicity, thereby deferring costly segregation and functional studies until phenotypic abnormalities arise. By restricting the aforementioned framework to families with affected male offspring, the protocol maintains ethical compliance, conserves resources, and focuses clinical efforts where they are most likely to yield actionable information. Successful implementation requires training pediatricians in DMD warning signs, establishing clear referral pathways, and integrating with existing newborn screening infrastructure. This model balances clinical utility with resource efficiency and may inform management of VUS in other X-linked disorders amenable to carrier screening, such as Fabry disease.

Combining NGS and MLPA for large-scale DMD carrier screening during the preconception and early pregnancy periods in populations such as Yueyang can effectively identify DMD carriers. This approach enables timely genetic counseling to ascertain any family history of DMD and guides at-risk individuals toward prenatal diagnosis, facilitating the early detection of potential genetic risks. Furthermore, continuous follow-up is essential for cases classified as P, LP, and VUS to gather additional clinical information. This practice helps accumulate phenotypic data under various genotypic conditions, thereby providing comprehensive information for subsequent genetic consultations.

Several limitations of this study should be acknowledged. First, as a single-center investigation conducted exclusively in Yueyang City, the generalizability of our findings to other populations remains to be validated. Second, despite the large initial screening cohort (n = 25,611), the absolute number of identified carriers (n = 28) was modest, which may limit the statistical power for subgroup analyses, particularly for the observed geographical heterogeneity. The high carrier rate in Yunxi District (0.74%) should therefore be interpreted with caution, as it may reflect random variation due to small numbers. Third, although we detected 81 VUSs, functional validation was not performed for the majority of them; the clinical correlation was demonstrated in only one prenatal case, making it premature to draw definitive conclusions about the clinical utility of VUSs. Fourth, our combined NGS and MLPA approach, while comprehensive for common mutation types, may have missed deep intronic variants, mosaic mutations, or structural rearrangements beyond the detection capabilities of these platforms. Fifth, while the combination of NGS and MLPA reliably identifies exon-level copy number variations, it does not permit precise determination of intronic breakpoints. The reading-frame rule applied in this study was therefore based on exon boundaries rather than nucleotide-level junction sequences, which is consistent with standard clinical practice for DMD CNV screening. Nevertheless, in cases where breakpoint-level resolution is required (e.g., unusual rearrangement patterns or recurrence risk refinement), additional approaches such as long-range PCR followed by Sanger sequencing or long-read sequencing would be necessary. Finally, no long-term follow-up was performed for non-carriers, preventing assessment of false-negative rates. Future multicenter studies with larger sample sizes and orthogonal validation methods are warranted to confirm and extend these findings.

## 5 Conclusion

This study provides the first comprehensive, population-scale characterization of DMD carrier frequency and variant landscape among women of childbearing age or in early pregnancy in Yueyang City, China, contributing the largest systematically screened variant dataset-including 28 P/LP variants and 81 VUSs, 12 of which (1 LP and 11 VUSs) were previously unreported-to a region where such data have been sparse. The observed carrier rate of 0.11% and the predominance of copy number variations, particularly deletions in the exons 45–55 hotspot region, align with known Asian mutation profiles while revealing substantial intra-regional heterogeneity. Importantly, our findings highlight the underappreciated clinical value of VUS in carrier screening: the identification of 81 VUSs, including one prenatally detected VUS associated with a postnatal clinical phenotype, suggests that a subset of VUSs may be pathogenic. We therefore recommend that VUSs, especially those with Bayesian prediction scores ≥3, be actively considered in genetic counseling and prenatal diagnosis rather than routinely disregarded. These results establish a critical evidence base for region-specific preventive strategies and underscore the need for refined VUS interpretation frameworks to further reduce DMD incidence.

## Data Availability

The original contributions presented in the study are publicly available. This data can be found at the Figshare repository, with the accession DOI: 10.6084/m9.figshare.30185923.v4.
